# Dilated Odontoma Arising in the Mandibular Third Molar Germ: Report of a Case of an Unusual Lesion in an Uncommon Site

**DOI:** 10.3390/diagnostics11122256

**Published:** 2021-12-02

**Authors:** Francesca Zara, Giacomo D’Angeli, Alessandro Corsi, Antonella Polimeni, Gian Luca Sfasciotti

**Affiliations:** 1 Department of Oral and Maxillo-Facial Sciences, Sapienza University of Rome, 00161 Rome, Italy; zarafrancesca94@gmail.com (F.Z.); antonella.polimeni@uniroma1.it (A.P.); gianluca.sfasciotti@uniroma1.it (G.L.S.); 2 Department of Molecular Medicine, Sapienza University of Rome, 00161 Rome, Italy; alessandro.corsi@uniroma1.it

**Keywords:** dilated odontoma, *dens invaginatus*, third molar, tooth germ, dental development

## Abstract

Dilated odontoma is the most severe variant of *dens invaginatus.* It is extremely uncommon in the posterior mandible. It is thought to originate during the morpho-differentiation stage of dental development. However, its etiology and pathogenesis remain obscure. We report here the clinical and pathologic findings of an incidentally discovered dilated odontoma arising in the left third mandibular molar germ of an 11-year-old male and a review of the pertinent literature. As dilated odontoma is not established as an independent entity in the current WHO classification of odontogenic tumors and is the result of a well-established developmental anomaly of the tooth (that is, the invagination of the enamel organ into the dental papilla), it should be better identified as dilated *dens invaginatus*.

## 1. Introduction

Dens *invaginatus*, also known as *dens in dente*, is a tooth developmental anomaly caused by an invagination of the enamel organ into the dental papilla. It may affect either the primary or the permanent teeth and its incidence has been estimated to range between 0.04% to 10%, with a greater female predilection [1]. The maxillary lateral incisors are the most frequently affected teeth, followed by maxillary central incisors, premolars, and canines [2,3]. The anomaly, which can be also symmetric, uncommonly involves the mandibular teeth, especially in the molar region [4]. The mostly used classification of *dens invaginatus* is that proposed by Oehlers, who distinguished three different main types based on the depth of enamel-lined invagination [5]. In type 1, the less severe and more frequent form, the invagination is confined to the crown. In type 2 and type 3, the invagination extends beyond the cemento-enamel junction either remaining within (type 2) or penetrating through (type 3) the root, leading in the latter case to the formation of an additional apical or lateral foramen. Swelling or pain related to a local periapical infection is the main clinical feature associated with *dens invaginatus* [3]. In rare cases, the invagination may significantly dilate, impairing the normal development of the involved tooth [2,3,6]. In this condition, which is commonly reported as dilated odontoma, the involved tooth shows a doughnut-like circular-oval shape with a radiolucent interior [2,3,6,7,8,9,10,11]. Thus, dilated odontoma is considered the extreme and most severe form of *dens invaginatus* [2,3,8]. 

We report here an unusual dilated odontoma incidentally detected in an 11-year-old male. The lesion was associated with the left mandibular third molar germ. A review of the pertinent literature, with a special focus on the extracted type III *dens invaginatus*, is also provided.

## 2. Case Report

**Figure 1:**
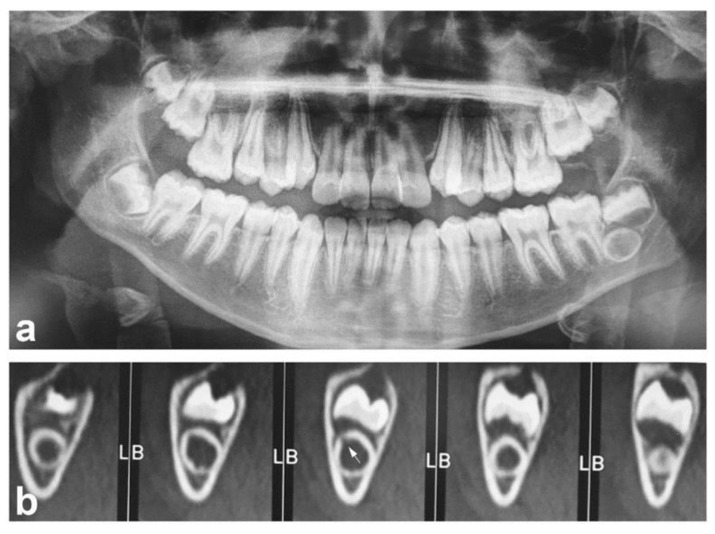
Panoramic dental radiograph (**a**) and CT scan (**b**) (L is for the lingual side and B is for the buccal or vestibular side) views of the lesion. It appears as an intraosseous doughnut-like lesion radiopaque at the periphery and radiolucent in the center, and is associated with the left mandibular third molar germ. The third frame of the illustrated CT scan shows a small radiolucency (arrow) suggestive of the invagination. CT was performed with a 64-slice CT scanner (Siemens Somatom Edge Plus 64, Siemens Helthineers, Germany). The acquisition was performed using the following parameters: tube voltage: 100 kVp; 13 mAs; rotation time: 1.0 s; delivered dose: 7 mGy; slice thickness: 1.25 mm; field of view: 160 mm.

**Figure 2:**
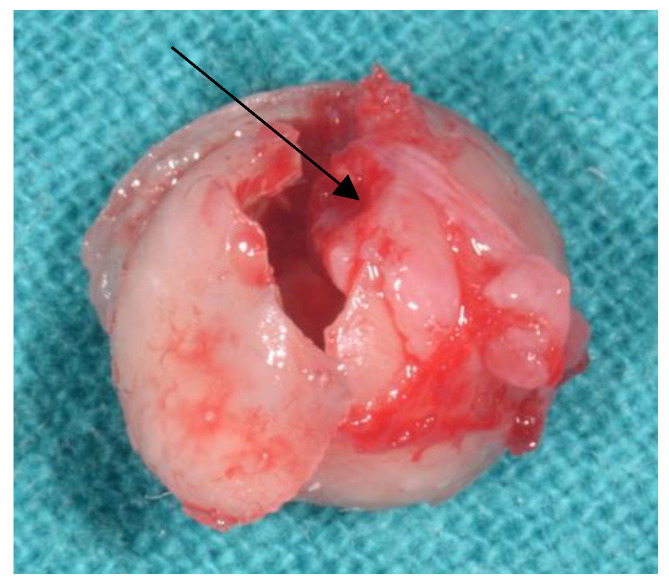
Gross appearance of the excised lesion. The walls of the lesion appear focally interrupted and no content is present inside. For orientation, the occlusal surface is indicated by the arrow.

**Figure 3:**
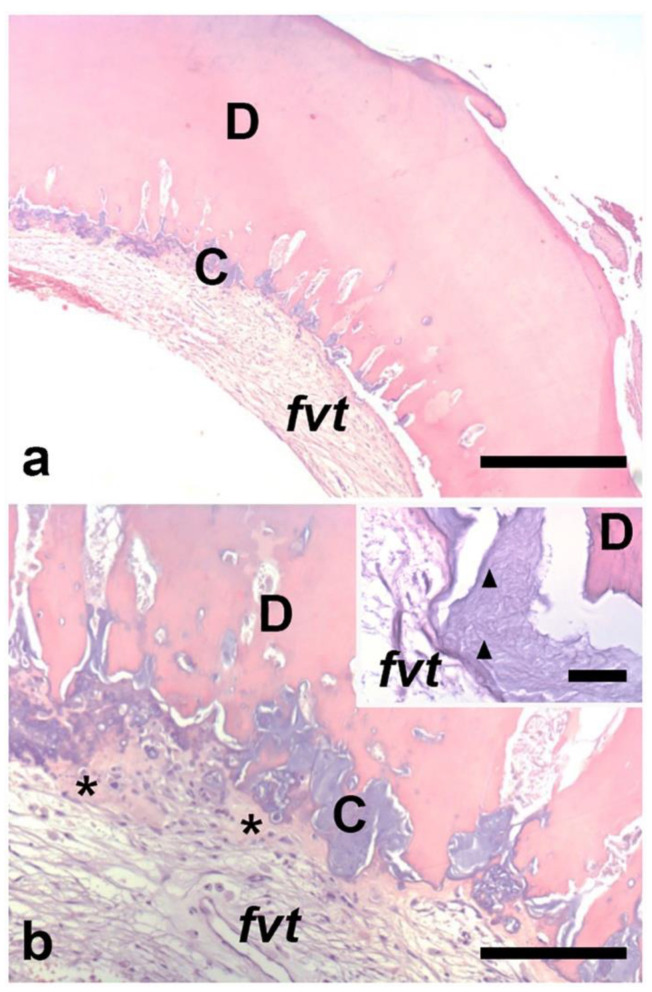
Low- (**a**) and high-power (**b**) magnification of the wall of the excised lesion. The outer wall is composed of dentin (D). The inner side of the dentin is strictly associated with a basophilic calcified dental tissue (C), which in turn is associated with an eosinophilic bone-like matrix (asterisks) at the interface with the inner fibro-vascular tissue (*fvt*). The insert in (**b**), in which the white clefts are artefactual, illustrates enamel with a rod-like structure (triangles) in the inner side of the dentin (D). Hematoxylin–eosin stain. Bars: 500 μm in a, 200 μm in b and 50 μm in the insert.

An 11-year-old male came to our observation for his first dental visit. His medical history was negative. No symptoms were reported by the patient or his parents. The face was symmetric and no swelling of the cervical lymph nodes was observed. Intraorally, the dentition of the permanent teeth was completed, except for the third mandibular molars and the second and third maxillary molars. Bucco-lingual expansion of the jaw bones was not evident. An orthopantomogram was performed to assess the development of third molars [12]. Unexpectedly, the analysis revealed an intraosseous doughnut-like lesion radiopaque at the periphery and radiolucent in the center associated with the left mandibular third molar germ (Figure 1a). Additional dental abnormalities were not observed. The maximum diameter of the lesion was 5.7 mm. Based on these findings, developmental abnormalities of the third molar (e.g., dilated odontoma) and odontogenic (e.g., cementoblastoma) and non-odontogenic (e.g., osteoblastoma or osteoid osteoma) tumors were considered for differential diagnoses. To better characterize the lesion, a computed tomography (CT) scan was required. The analysis established bone integrity around the lesion and its independence from the local neuro-vascular structures. In addition, it revealed, on the sagittal projection, a small gap in the proximity of the buccal surface of the mandible (Figure 1b). As the most significant clinical concern related to this condition is the risk of developing pulpal necrosis, it was decided to extract the germ of the third molar and the underlying lesion. To do this, under local anesthesia, a mucoperiosteal flap was raised posterior to the mandibular right second molar. The vestibular cortical plate was removed, exposing the ovoid mass, which was removed with the germ of the mandibular tooth. The surgical flap was repositioned and sutured. Healing was uneventful. The excised lesion appeared as an empty hard spherical mass virtually devoid of content (Figure 2). It was routinely processed for paraffin embedding after fixation and decalcification. Histologically (Figure 3a,b), the outer hard tissue was dentin. The inner part of dentin was in continuity with basophil calcified material, which in turn was focally in contact with the bone-like matrix. The basophil calcified material focally presented a rod-like structure consistent with enamel (Figure 3b, insert). More centrally, the lesion was composed of fibro-vascular tissue. The pathologic findings were considered as consistent with a dilated odontoma.

## 3. Discussion

We have presented here a rare incidentally discovered dilated odontoma arising in the left third mandibular molar germ of an 11-year-old male. To the best of our knowledge, dilated odontoma has never been reported to arise in association with the germ tooth. This makes the case reported here unique. In the most recently published review of the literature, sixteen extracted type III *dens invaginatus* cases were reported [3]. Eight of these cases were classified as dilated odontoma [1,2,3,8,9,10,11,13], while the other four cases were published by Matsumoto and Seto [14]. A clinical synopsis of these cases is reported in Table 1. In four adult patients (three females and one male, age range of 28–60 years), the dilated odontoma involved the third molar region [8,9,14]. In addition to the case reported here, the dilated odontoma was detected incidentally in two cases [3,8].

In addition to its clinical uniqueness, the case reported here offers the possibility to discuss the proper terminology to use for this type of dental lesion. In this regard, we completely agree with other authors, according to which dilated odontoma represents the extreme and most severe form of type III *dens invaginatus* [2,3,8]. However, in our opinion, as also recently suggested by others [15], dilated odontoma is a misnomer and this terminology should be abandoned because it tends to be confusing. Dilated odontoma is certainly a hamartoma in the sense that it reflects a defect (“hamartia”) in the development of a tooth (i.e., the invagination of the enamel organ into the dental papilla before the mineralization of the dental tissues begin) but it cannot be viewed as a tumor-like hamartomatous growth composed of a mixture of dental hard and soft tissue, as odontomas are [15,16,17]. Indeed, this invagination leads to the development of morphologically abnormal teeth and the expected final is the formation of a doughnut-like spheric or ovoidal calcified structure with a more radiolucent central portion [2,3,5,8,9,10,11,13]. This view is also strongly supported by the current WHO classification of odontogenic tumors, whereby dilated odontoma is not established as a specific entity in the general spectrum of the odontogenic tumors and within the specific context of the odontomas [17]. For these reasons, we feel that the term dilated *dens invaginatus* or dilated hamartoma of the teeth to identify these lesions could be more accurate than dilated odontoma.

Different theories have been suggested to explain the development of *dens invaginatus* or dilated odontoma. Although there is general agreement on its origin during the morpho-differentiation stage of dental development, as unequivocally proven in the case reported here, the fine etiology and pathogenesis remain to be elucidated. Proposed theories have included increased localized external pressure, inner enamel epithelium growth retardation or stimulation in certain areas of the tooth bud, infection, and trauma [2,3,5,6,7,8]. Genetic factors have also been considered but they were excluded in our case because the lesion was isolated and the clinical and radiographic evaluation failed to reveal other dental anomalies [18].

In conclusion, the case reported here represents an uncommon example of severe type III *dens invaginatus* or dilated odontoma incidentally detected in an 11-year-old male. The lesion involved the left mandibular third molar germ. Even though the etiology and pathogenesis of this lesion remain to be defined, its origin reflects a well-defined developmental anomaly in tooth formation that is the invagination of the enamel organ into the dental papilla. As a consequence, although the existing nomenclature is more clinically acceptable, it could be simply defined as dilated *dens invaginatus* or dilated hamartoma of the teeth.

## Figures and Tables

**Table 1:** Clinical synopsis of the patients with extracted dilated odontoma reported in the literature.

Age/Gender	Clinical Presentation	Tooth Number	Ref.
24 years/Male	Pain and swelling	2.1	[1]
14 years/Female	Unerupted molar tooth	3.7	[2]
7 years/Female	Asymptomatic	4.2	[3]
28 years/Female	Asymptomatic	4.8	[8]
47 years/Female	Pain and discomfort	3.8	[9]
16 years/Male	Swelling	4.4 bis	[10]
24 years/Male	Pain and swelling	1.7	[10]
18 years/Male	Malformed tooth and intermittent mild pain	2.1 bis	[11]
14 years/Male	Swelling	2.3	[13]
60 years/Male	NA	3.8	[14]
30years/Male	NA	4.6	[14]
49 years/Female	NA	1.8	[14]
32 years/Female	NA	right maxillary molar region	[14]
11 years/Male	Asymptomatic	3.8 (germ tooth)	Present case

Adapted from Table 1 from [3]. NA: not available; bis: supernumerary tooth.

## Data Availability

Data are available from the authors upon reasonable request.
